# Understanding radiographic decision‐making when imaging obese patients: A Think‐Aloud study

**DOI:** 10.1002/jmrs.543

**Published:** 2021-09-08

**Authors:** Grace Seo, John Robinson, Amanda Punch, Yobelli Jimenez, Sarah Lewis

**Affiliations:** ^1^ Discipline of Medical Radiation Sciences, Faculty of Medicine and Health The University of Sydney City Road Camperdown NSW Australia

**Keywords:** adaptive technique, education, obesity, obesity decision‐making, radiography

## Abstract

**Introduction:**

The incidence of obesity has been steadily rising over the last few decades and is having a significant impact upon the health system. In radiography, a particular challenge of imaging obese patients is implementing the as low as reasonably achievable (ALARA) principle when determining radiation dose, and technical and patient‐care adaptations. This study aimed to better understand the decision‐making strategies of experienced radiographers in determining imaging and exposure factor selection in the context of imaging obese patients.

**Methods:**

This study employed a ‘think‐aloud,’ methodology, and eight experienced diagnostic radiographers working in clinical education were recruited to perform routine AP abdominal X‐ray projections on an anthropomorphic phantom. They were asked to simultaneously verbalise emerging thoughts as they considered positioning, exposure selection and image evaluation. This process was repeated with three different phantom sizes, each representing an increased BMI from ‘healthy,’ to, ‘morbidly obese.’ Audio recordings were transcribed and interpreted via Bowman’s (1997) theory of radiographic judgement and decision‐making.

**Results:**

Analysis of interview transcripts identified 12 key concepts considered by experienced radiographers. Differences in radiographic concepts were considered when imaging phantoms of different sizes was demonstrated. A shift from segmental (e.g. positioning) to more environmental factors (e.g. patient comfort) and an increase in the number of verbal considerations with increasing phantom size were identified. The shift in focus of decision‐making stages identified the greater need to consider contextual factors such as patient comfort and repeatability when imaging obese patients.

**Conclusion:**

Experienced radiographers find imaging obese patients challenging and alter their perception of image quality to accommodate for patient presentation. The findings will help inform future research, practice guidelines and learning resources to provide optimal imaging and care for obese patients, especially for student education.

## Graphical abstract

Radiographers' decision making when imaging obese patients is complex and involves assessing a range of factors, such as technical, environmental and the holistic nature of the examination. Using a Think‐Aloud technique, we examined the judgement of expert radiographers to imaging an obesity phantom. As the phantom increased in size, radiographers were less likely to consider technical factors and changed their decision‐making to considerations of the holistic nature of the examination.

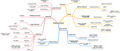

## Introduction

### Obesity and radiography

Obesity is the biggest contributor to the non‐fatal burden of disease in Australia, with 67% of Australian adults classified as obese or overweight in 2018.^1^ Obesity is currently defined by the World Health Organisation (WHO) as having a body mass index (BMI) of 30 or above.^2^ The incidence of obesity is expected to increase over the next decade, and its association with multiple chronic conditions renders it arguably one of the most prominent health risk factors in the modern society.^1^, ^3^ This demographic shift is also reflected in the patient population, creating a need for increased awareness and understanding of the obesity epidemic in order to provide equivalent quality healthcare.^4^, ^5^ This is especially important in radiography which serves as the primary diagnostic modality for many patients.^6^, ^7^

The limitations of current radiographic practice in catering to patients of increased body habitus are already apparent. Image acquisition and interpretation have become compromised with one study observing the number of ‘habitus‐limited,’ radiological reports doubling over a 15‐year period.^5^ Adaptations to technique must be considered when imaging obese patients, which may include changes to patient positioning, transportation, tactile identification of anatomical landmarks, equipment choice and determining exposure parameters for obtaining images of diagnostic quality.^8^, ^9^, ^10^, ^11^

Although adaptive technique is considered standard practice, there is a lack of available obesity education resources, resulting in reduced strategies to guide these modifications. Thus, obese patients are often subjected to repeated projections and subsequently increased radiation dose.^12^ As a result, suboptimal images may be accepted for diagnosis in a quasi‐adherence to the as low as reasonably achievable (ALARA) principle, having potentially adverse implications for diagnosis and patient treatment.^4^, ^5^

### Optimising exposure parameters

There is no safe limit of ionising radiation dose, and thus, obtaining optimal diagnostic images at minimal dose should form the basis of all radiographic procedures.^13^, ^14^ Adhering to this principle is particularly challenging in the context of obese imaging. The comorbidities associated with obesity often means obese patients undergo more diagnostic imaging than non‐obese patients, and current limitations of radiographic equipment size may require multiple views of each projection to cover the region of interest.^5^, ^8^, ^12^ Thus, obese patients experience a greater average stochastic risk, rendering optimisation of radiation dose for this patient population of utmost importance.^15^, ^16^

Studies have shown that although this elevation of exposure for obese patients is considered common practice, there is an evident lack of standardised implementation, reflecting the gap in understanding these parameters.^17^

Most of the adaptations are based on comparisons made with the, ‘average,’ patient thickness which is subjective to the population, creating variability in exposures of up to 25%.^12^, ^13^ Most strategies were also developed for film‐screen radiography, which has now been widely replaced with computed radiography (CR) or direct‐digital radiography (DR), and there has been little research in modifying these techniques for the modern digital systems.^18^, ^19^

Although errors in underexposure are identifiable due to noisy images, overexposed images can often be manipulated with post‐processing to go unnoticed.^20^ A study by Uffmann & Schaefer‐Prokop^21^ reported that radiographers tended to increase exposure factors when unsure of the patient’s size.

Some dose optimisation measures specific to DR systems have been introduced in an effort to avoid consistent overexposure. Although these DR techniques show potential for dose reduction, they face similar limitations as the film‐screen strategies. Most of these measures fail to account for larger patient sizes across different projections and lack standardisation between manufacturers and institutions.^22^, ^23^, ^24^

### Decision‐making strategies

The difficulty in establishing a standardised approach to adaptive technique demonstrates the importance of understanding radiographic decision‐making at the individual level. Although protocols exist as a method of standardisation, often it is only the routine projections for average‐sized patients (BMI 19‐24) that are outlined. These radiographic decisions are often complex and made in high‐pressure settings, requiring quick judgement and action based on the unique clinical situation.

The limited studies of decision‐making in radiography have found that experienced professionals make rapid judgements in an intuitive manner.^25^, ^26^ This can be defined as making choices with ‘an immediate understanding of knowledge, combining instinct and intelligent thinking’.^27^ Clinical reasoning for experienced health practitioners appears to be based on experience and is highly subjective, varying with individual expertise, philosophy and training background with one study stating that ‘the best decisions are made intuitively by those with the most experience’.^25^

This reliance on experience and intuition to form clinical judgements can be a source of frustration for students as these skills are difficult to formally teach. Clinical placements are highly valued by students as they present with opportunities to gain experience while under the supervision of clinical educators who can impart practical knowledge and expertise.^28^ Radiographic teaching in the university setting leans towards theory‐based education, which can be attributed to the limitations in what can be demonstrated and taught outside of the clinical setting through simulation alone. This constraint is further accentuated in the context of adapting technique when imaging obese patients as simulation equipment tends to represent the, ‘average‐sized’ or lean patients.^28^

Radiography student practitioners have reported feeling anxious and inadequate, especially when working with obese patients whom present challenges that mandate quick clinical decision‐making.^10^, ^28^ Thus, a gap between the present patient demographics and available resources for educating radiographers in adaptive technique is identified.

## Research aim

This study aimed to explore the decision‐making strategies of experienced radiographers in determining imaging and exposure factor selection in the context of obese patients. Currently, little is known about the individual cognitive steps taken in the decision‐making processes of radiographers when adapting technique for obese patients.^11^, ^14^ Breaking down intuitive decisions into individually explored thought processes could help facilitate the development of learning resources through the identification of key factors influencing the decisions radiographers make.

## Methods

This study received ethics approval from the Human Research Ethics Committee (HREC) at the University of Sydney (Approval #2019/810). Participation was voluntary, and written informed consent was obtained from all participants.

### Think‐Aloud Methodology

A Think‐Aloud (TA) methodology was employed in this study to investigate the cognitive processes of experienced radiographers. Radiography is often classified as a human technical science encompassing technical imaging and patient interaction skills.^29^ Thus, research methodology should address both these factors when aiming to understand what influences radiographic decision‐making. TA methodology requires participants to verbalise emerging thoughts while performing a set task.^30^ Verbalisation requires individuals to draw upon their working memory, which acts as a processing unit between short‐ and long‐term memory.^31^

### Sample

As the aim of the study was to obtain information‐rich data from experienced radiographers, purposive sampling was employed. Inclusion criteria required participants to have a current role in clinical education, a minimum of five years of clinical experience and general registration with Medical Radiation Practice Board of Australia (MRPBA). Exclusion criteria consisted of radiographers who were unable to travel to the university setting to complete the research task. To satisfy these criteria, a voluntary recruitment email was distributed to diagnostic radiographers currently affiliated with the University of Sydney Work Integrated Learning (WIL) or academic program. A final sample size of eight experienced diagnostic radiographers was recruited, considering theoretical saturation on a number of key concepts.

### Think‐Aloud Interviews

All TA interviews were conducted at the University of Sydney in the CARESTREAM DRX Direct Radiography facility by one researcher. The interviews were conducted individually over four sessions spanning 3 months from January 2020 to March 2020. An audio recording device was worn at the chest level of participants to obtain audio recordings for transcription and analysis. A pilot of the study protocol was conducted by one participant who did not take part in the main round of data collection. Small changes were made to the study preparation prior to formal data collection commenced, including dressing the phantom in clothing to simulate a patient gown and preparing the machine for exposure.

The TA interviews began with a set of structured interview questions to obtain participant demographic data as well as familiarise individuals with the interview style. These data were preliminary and only used to orientate the participants and check they were within the inclusion criteria. Participants were then asked to perform a routine anteroposterior (AP) abdominal X‐ray on a Kyoto Kagaku PBU‐60 anthropomorphic phantom (Kyoto Kagaku Pty Ltd, Kyoto, Japan) so as to simulate a clinical environment. This projection was chosen as it commonly performed and presents with challenges to dose optimisation, specific to the context of imaging obese patients such as increased body circumference and concentration of adipose tissue. When compared with other bodily regions, plain abdominal imaging showed the most significant increase in radiation dose received by obese patients than that of the average‐sized patient.^15^

Verbal instructions were given to participants to articulate their clinical decision‐making process as they considered:

- Positioning
- Exposure parameter selection and
- Image evaluation

**Figure 1:**
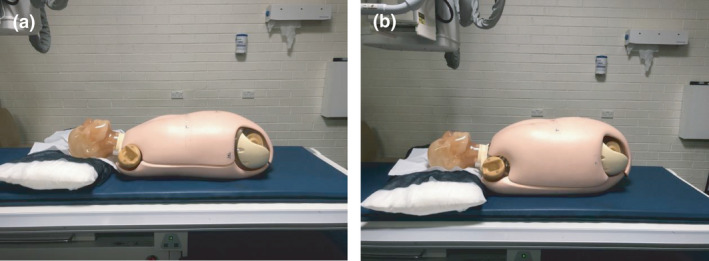
Think Aloud Interview Set‐up of Phantom with BMI 32 and BMI 40 Adipose layers (a) left and (b) right (a) shows phantom with additional layer to make BMI 32 simulation (b) shows phantom with additional layer to make BMI 40 simulation.

Participants irradiated the phantom and produced radiographs for evaluation for each task. This task was repeated over three different phantom sizes, with task one being the standard BMI radiography phantom (BMI 18,5), and tasks 2 and 3 each representing an increased BMI (32 and 40). The phantom was built up with body plates that simulated adipose tissue and covered with a patient gown, as seen in Figure 1.

In accordance with TA methodology, interactions with the participant were kept to a minimum to avoid disruption of focal thought elicitation.^30^ Participants were only interrupted to prompt verbalisation after a prolonged period of silent work or to explain task requirements when participants were unsure. Semi‐structured interview questions were asked retrospectively to clarify responses that were ambiguous or lacked detail to aid the transcription process. The TA process for each participant was approximately 1 hour.

### Analysis

**Figure 2:**
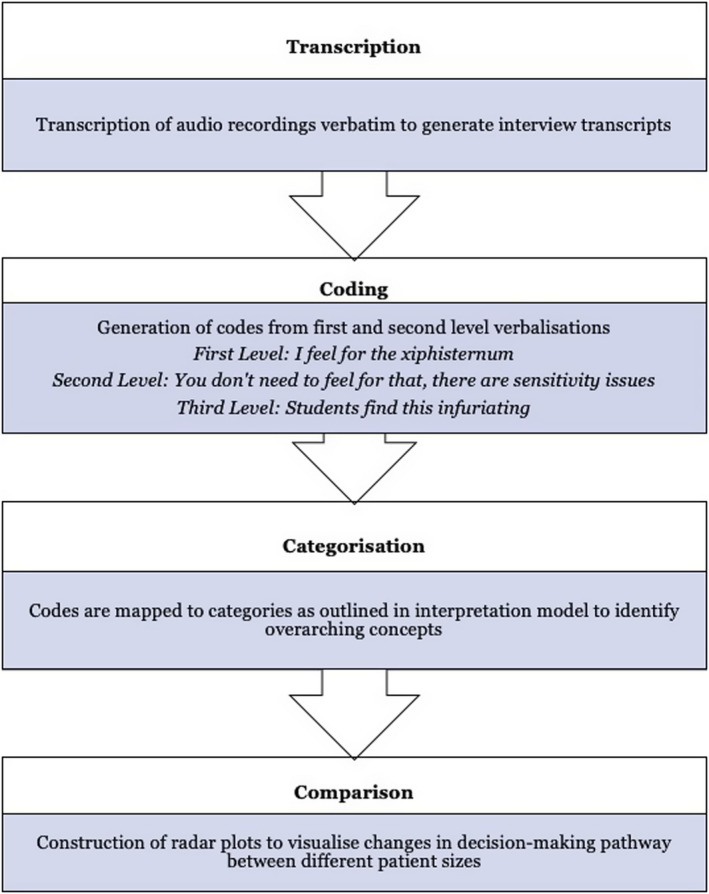
Analysis process with some examples of levels.

Data analysis was conducted following Ericsson & Simon’s^32^ TA methodology in four key steps as outlined in Figure 2. It is important to note that the aim of TA analysis is not to judge how well a participant has performed the set task but rather to record the thinking path of the participant in order to gain an insight into their decision‐making process.

The TA interview audio recordings were transcribed under intelligent verbatim rules by one researcher to generate interview transcripts.^33^ Having the interviewing researcher (who is a senior radiography student) transcribe the audio recordings was an important strategy in improving transcript quality and accuracy and ensured understanding of the technical terms used.^34^ Code names, such as ‘R1’, were assigned to participants, and identifying features such as names of individuals and institutions were removed to maintain anonymity. A member check of the transcript was provided to 50% (n = 4) of the participants who each confirmed that this was an accurate representation of their TA data.

Line‐by‐line encoding was applied to the transcripts to generate codes inductively, based on the information contained in each segment of verbalisation. Segments of verbalisation correlated to sentences, clauses or single words that contained one idea. Three levels of verbalisation are identified in TA analysis. The first level pertains to simple statements that do not require further thought before articulation. Most verbalisations at this level can be directly encoded. Second‐level verbalisations contain more abstract concepts that require additional explanation. Participants may require more time to process thoughts before verbalising them, but focal information remains the same. Finally, verbalisations at the third level have been further processed and draw from the long‐term memory, rather than the immediate working memory, which is accessed for decision‐making (Lundgren‐Laine & Salantera^31^). At this level, verbalisations are no longer relevant to the task being performed. Examples of the levels of verbalisation are included in Figure 2.

As per Ericsson & Simon’s^32^ TA methodology, only first‐ and second‐level verbalisations were considered. It is recognised that verbal gaps in data may occur due to several reasons such as participants finding difficulty in articulating thoughts or some thought processes being omitted because of the rapid rate at which they occur. For this reason, methodology suggests an interpretation model is employed in guiding analysis in a confirmatory manner.^32^, ^35^

Bowmans’ theory of radiographic judgement and decision‐making for plain radiographic examinations (1997) was chosen for this study as it is a known interpretation model from previous research. Bowman^25^ identified three stages of decision‐making in radiographic practice: segmental, holistic and environmental. The segmental stage relates to the consideration of individual technical elements of a radiograph. This includes positioning, quality control measures and image evaluation to a tentative point of acceptability. The holistic stage considers the overall combination of the individual elements on a scale of acceptability. The final order of judgement is the environmental stage where factors such as patient condition and time of day are considered.

**Figure 3:**
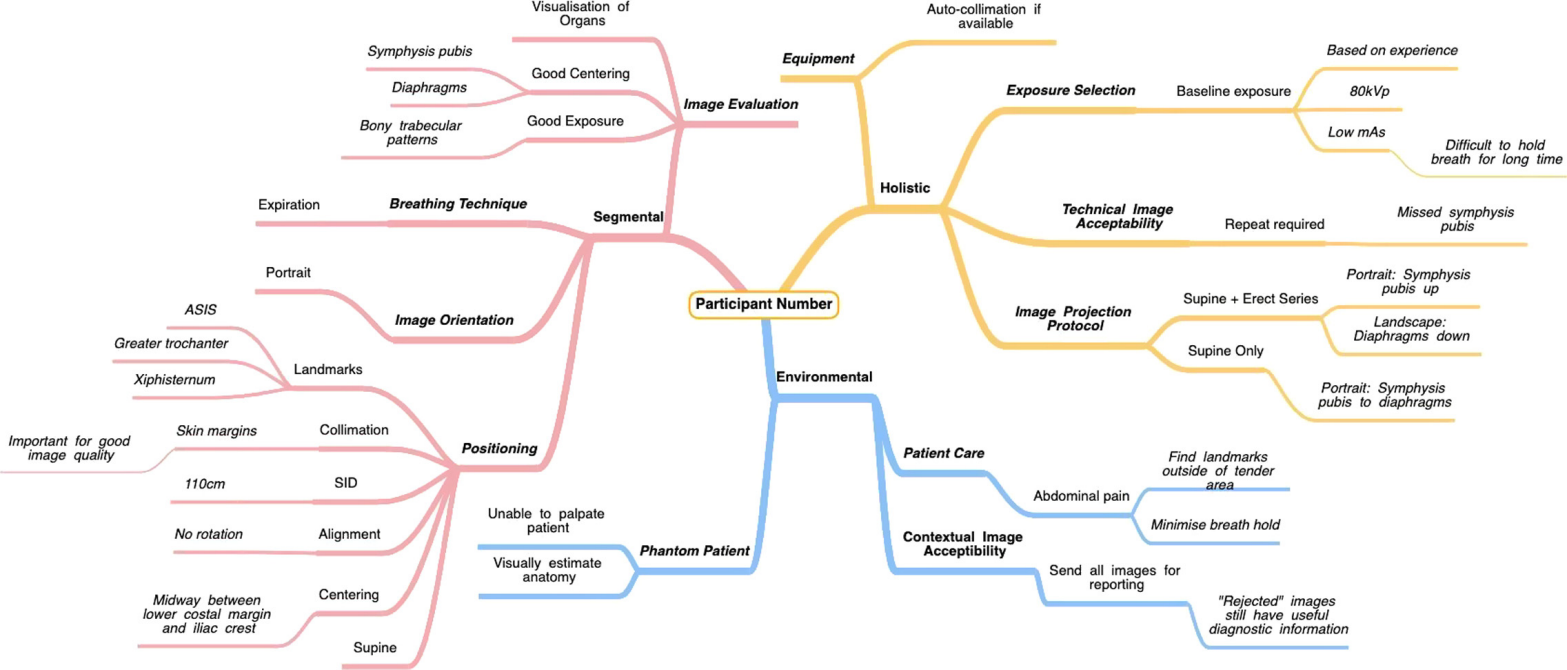
Concept map for decision making for a participant.

The generated codes were mapped to these stages of radiographic decision‐making and grouped to visualise emergent themes for each task performed. A concept map of decision‐making was constructed for each participant and for each task (see Figure 3 for an example), with a total of 24 concept maps developed. A review of these concept maps identified the overarching salient concepts which were discussed by all researchers before being finalised. The frequency of the verbalisations for each concept were then tallied and used to construct decision‐making webs to best visualise the fluid cognitive processes of participants when considering technique and strategy in imaging obese patients.

## Results

A total of 12 key concepts were identified from the concept maps with four in each decision‐making stage (Table 1).

**Table 1:** Key concepts and decision‐making stages.

Key concept	Definition
*Segmental stage*	
Positioning	Technical factors: centering, collimation, alignment etc.
Image orientation	Orientation of cassette: landscape vs portrait
Breathing technique	Patient instructions in regard to breath hold
Image evaluation	Acceptability of individual image technical components
*Holistic stage*	
Image projection protocol	Outline of images to be taken to cover region of interest
Exposure selection	Exposure technique and parameters chosen: kVp, mAs
Landmark adaptation	Alternative landmarks or methods in gauging anatomy
Technical image acceptability	Acceptability of image as a combination of its elements
*Environmental stage*	
Patient care	Adaptations considering patient welfare and comfort
Larger patient expectations	Anticipation of challenges associated with larger patients
Radiographer confidence	Uncertainty regarding decision being made
Contextual image acceptability	Acceptability of image in the context of imaging reason

**Figure 4:**
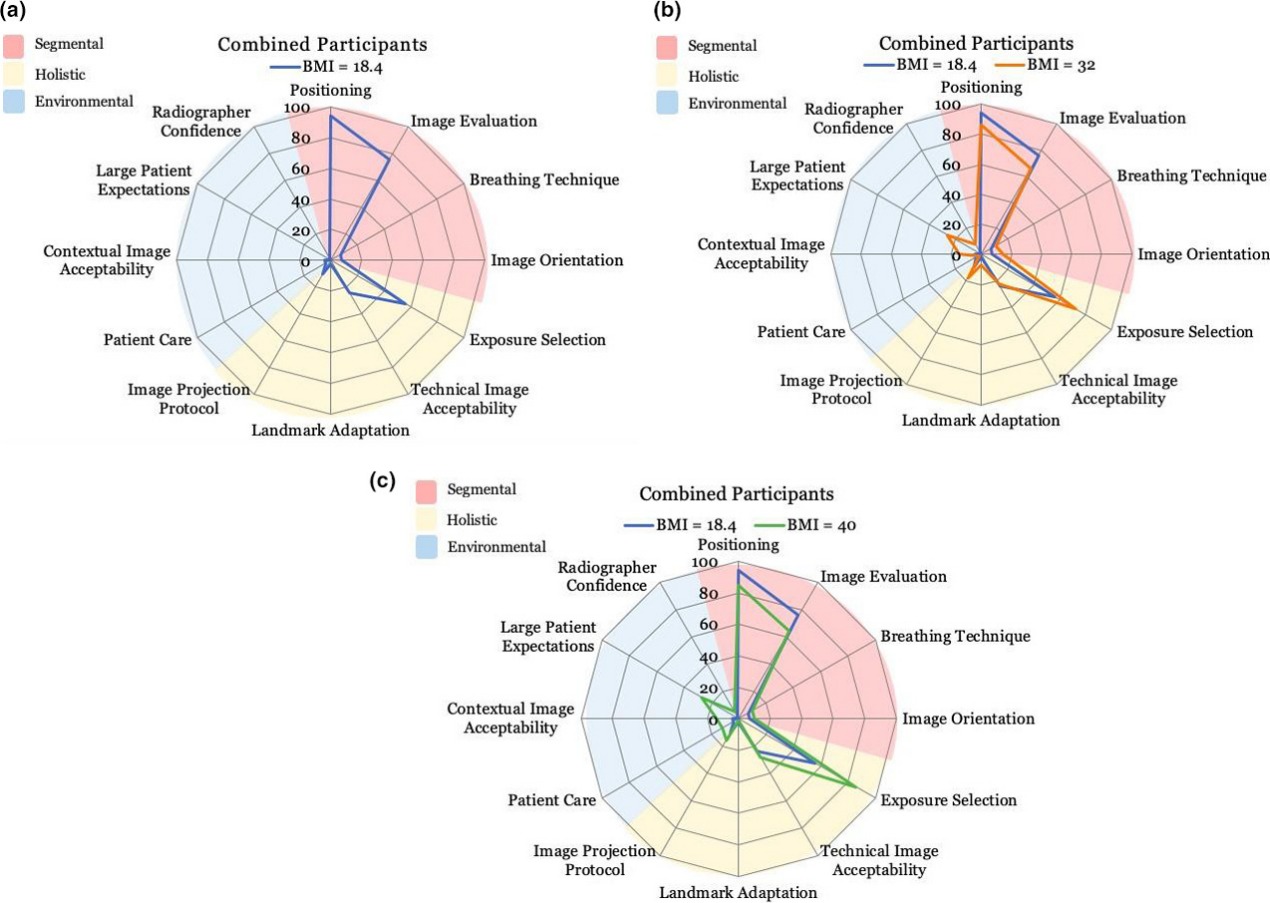
(a) Decision web of combined participants task 1 (BMI 18.4) (b) Decision web of combined participants for task 1 (BMI 18.4) & task 2 (BMI 32) (c) Decision web of combined participants for task 1 (BMI 18.4) & Task 3 (BMI 40).

Decision‐making webs were then constructed to reflect frequency of the key concepts identified through the TA interviews. The area within the plot does not represent ordinal data but rather visually represents the direction of key concepts verbalised during the three tasks. The webs can be used to visualise changes in radiographer decision‐making when viewed across the different patient sizes. The numbers ranging from 0 to 100 in Figures 4a to 4c represent the combined total of verbalisations mentioned for each concept.

### Imaging of the Average‐Sized Phantom (BMI 18.4)

A strong focus on segmental factors can be identified when participants imaged the average‐sized phantom (Figure 4a). The majority of the participants’ verbalisations were concerned with concepts such as patient positioning and evaluation of the individual elements of the radiograph. These concepts formed the bulk of verbalisations for Task 1.

R7: ‘I’d check the tube is centered. Then I tend to center in the midline, midway between.. at the level of the iliac crest in the midline’.

The majority of verbalisations in this category were concerned with establishing an exposure technique and a threshold of technical radiograph acceptability.

R6: ‘I’d use 70kV. That’s what I start off with. I run a fixed kV and I alter my mAs. Very rarely will I alter mAs and kVp at the same time’. R3: ‘I would repeat this because we haven’t got the bladder on for Information’.

There was minimal mention of environmental concepts in decision‐making.

### Imaging of the Obese Phantom (BMI 32)

When imaging the obese patient, consideration for segmental concepts decreased and a growth in the holistic and environmental stages of decision‐making was observed (Figure 4b). In the environmental stage of decision‐making, participants began considering image acceptability on a contextual scale and verbalised expected differences in patient presentation associated with larger patients.

R5: ‘The larger the patient, the more likely the bowel is going to be expanded. You would certainly consider that you wouldn’t necessarily get it [anatomy] all on’.

R2: ‘When you start to get those larger patients and then it’s obviously going to be grey anyway… but you’re looking for soft tissue things aren’t you’.

An increase in participants stating their lack of confidence during image acquisition was also noticed. This uncertainty was most commonly expressed in relation to patient positioning and determining exposure technique.

R6: ‘Obese patients do make me nervous because it’s really hard to judge where everything is’.

Holistic concepts were also more frequently considered when participants were asked to image the obese phantom, and this is visualised as growth in the number of statements regarding exposure selection, landmark adaptation and projection protocol. These verbalisations were mostly in relation to common adaptive techniques required in radiographic imaging of obese patients.

R2: ‘I’d say if there’s a BMI of sort of 32, that’s a really obese patient, I would generally be using two shots in landscape’.

R4: ‘The average difference between the lower costal margin and iliac crest is only a few centimetres so I can use that as a guide to try and push a little harder around the iliac crest region’.

Although less than the imaging of the average phantom, the volume of verbalisations regarding segmental factors remained the highest in frequency and demonstrated a similar distribution of concepts as Task 1.

### Imaging of the morbidly obese phantom (BMI 40)

The shift towards environmental and holistic stages of decision‐making is further evident when comparing imaging of the average and morbidly obese patient (phantom size BMI=40), (Figure 4c). Similar to the imaging of the obese patient, a decrease in segmental concepts such as positioning and image evaluation can be observed. The consideration of image acceptability on a multifactorial level, technically and contextually, was also similar between Tasks 2 and 3.

The growth in consideration of exposure selection is greatest in the holistic stage of decision‐making. Participants often had to reassess their exposure technique, taking into account changes made to positioning due to the larger patient size.

R1: ‘Now, because I’ve increased the height as well as the patient being larger, I want to do that… I think that’s how I’m going to alter that, so I’ve gone up 10kVp’.

R7: ‘I’m just mindful that although that’s the upper limit for contrast, my concern is it won’t be penetrating enough so I’ll have to give the beam enough to get through what could potentially be a lot of tissue’.

A general increase in the number of verbalisations made in the environmental stage of decision‐making was identified. There is marked growth in frequency of considerations related to patient care, with participants accounting for possible limitations in patient mobility and demonstrating evidence of consideration for ways to make the projection more comfortable for the patient.

R5: ‘Sometimes large patients are awkward in their movements and so it can be a little bit hard for them to adjust that. You need to allow them a little bit more time to be able to get into the position you require them to be in’.

### Decision‐making process

From the decision‐webs, an overall change in the considerations made as the patient size changed was visualised. With increasing patient size, a shift in focus from segmental elements to more holistic and environmental factors was observed. Image acceptability was seen to be considered on multiple levels when imaging larger patients and the total number of verbalisations increased with patient size. It was also noted that not every participant considered all 12 key concepts in their decision‐making process, however verbalisations in all three stages of decision‐making: segmental, holistic and environmental, were identified for all eight participants.

## Discussion

This study reveals new knowledge of the decision‐making processes of experienced radiographers when undertaking a simulated activity to replicate imaging of obese patients. The TA methodology allowed for identification of key concepts, and there were demonstratable changes in considerations in response to different patient sizes.

When imaging the average size phantom, the verbalisations were concentrated in the segmental category. Concepts explored in this stage of decision‐making are primarily concerned with the individual technical elements that form the image, which are formally taught in the university setting.^28^ These segmental concepts form the basis of routine radiographic practice, and there was little variation in the codes generated between radiographers at this level. This suggests a level of standardisation exists at this stage of decision‐making, reflecting the applicability of ‘textbook technique’ for this patient group. It is interesting to note that despite the apparent simplicity of this stage in decision‐making, the total number of verbalisations for segmental concepts was greatest for all three phantom sizes. This demonstrates the deeply complex nature of these segmental decisions that are made rapidly by radiographers in common practice.^25^

Experienced radiographers made more considerations and verbalisations in the holistic and environmental stages of decision‐making than that of the average‐size phantom. This shift in focus may be due to the numerous adaptations to standard technique required in imaging obese patients. Although segmental concepts may form the technical basis of radiographic procedures, the holistic and environmental stages of clinical reasoning encompass the humanistic aspect of radiography that must be considered to address the unique needs of the patient. This can be seen in the increased volume of thoughts related to concepts such as patient care and expectations of challenges related to image quality that larger patients will likely present.

Adaptive technique requires the radiographer to draw on knowledge that is not systematically taught, resulting in highly intuitive decision‐making that varies on an inter‐radiographer basis due to differences in personal experiences.^10^, ^27^ This is suggested in the increased variation of generated codes for the holistic and environmental concepts in this study, echoing findings from van den Heuvel et al.,^17^ who found that when imaging obese patients, experienced radiographers had greater difficulty in reaching consensus for the humanistic aspects than for technical decisions such as positioning. Furthermore, our study concurs with the van den Heuvel et al.^17^ study in demonstrating that experienced radiographers placed a significant focus on patient care, with unique considerations for the physical comfort of obese patients.

The overall increase in the number of verbalisations with increased phantom size across holistic and environmental concepts reflects the increasing complexity of the task. This is exemplified in the consideration of image acceptability. When imaging the average‐sized phantom, the image acceptability was mainly evaluated in terms of its technical elements and its holistic value, such as if the anatomy was depicted in a standard visual format, for example, bladder at the bottom of the image. The verbalisations saw that when the phantom size increased, radiographers altered their evaluation of image acceptability on a new contextual scale of acceptance, introducing factors such as patient immobility, suspected pathology and limitations to inherent contrast. Most of the experienced radiographers expressed feelings of uncertainty towards making technical decisions when the phantom size reached BMI 40, especially during exposure selection and identification of landmarks. Although this lack of confidence in regards to imaging obese patients is recognised amongst novice radiographers, it is not usually associated with experienced radiographers.^36^ Although experienced radiographers were able to demonstrate a level of intuitive decision‐making in this study, further practice and training in obese imaging could supplement the development of intuitive judgement to increase confidence.

Multiple participants commented on the difference in tactile feel of the anthropomorphic phantom in comparison to the real‐life patient, expressing difficulty in accurately translating their skills to the task at hand. Although this may have complicated the task for participants, the loss of tactile orientation is a common challenge to the imaging of obese patients, demonstrating its relativity to this study.^8^ Participants often questioned the rationale for the imaging request, asking for the clinical history, symptoms and pathological queries of the phantom patient. This highlights the deeply ingrained principle of justification in the use of ionising radiation and reinforces the importance of contextual considerations in radiographic decision‐making identified in this study, which should be considered in future research.^13^, ^14^

Future research should also continue to pursue an understanding of radiographer decision‐making in the context of obese imaging, focusing on concepts found in the holistic and environmental stages which demonstrated the greatest variation. It would be of interest to recruit participants who work specifically in the bariatric clinical setting to glean further insights into commonly implemented strategies in imaging this patient demographic. It would also be interesting to compare the decision‐making processes of student radiographers who lack the experiential knowledge of experienced radiographers to observe the process of intuitive reasoning development.^28^

Although information‐rich data exploring the radiographic decision‐making of experienced radiographers when imaging obese patients were obtained in this study, several limitations exist. Due to the limited time frame in recruitment, collection and COVID‐19 social distancing requirements, participants were sampled from a defined geographic region; hence, the final sample size was smaller than anticipated. Furthermore, previous TA studies in radiography with similar sample sizes have been found to have reached theoretical saturation. It is important to note that this study is novel in combining TA methodology with a complex practical radiographic activity, whereas previous TA studies such as Yoon et al.^37^ have reported theoretical saturation with a study involving a non‐practical (image interpretation) task.

## Conclusion

This study provides new insights into radiographic decision‐making when imaging obese patients. Via a TA methodology and concept maps, the data explored experienced radiographic judgement to identify key concepts considered when imaging obese patients and observed an overall shift of focus from segmental to more holistic and environmental considerations with increasing patient size. The greater complexity of decision‐making in adaptive technique when imaging obese patients was further identified in the increased volume of verbal considerations and an articulated reduction in participant confidence when presented with an obesity phantom of BMI 40. These findings will help inform the formulation of new evidence‐based practice guidelines and learning resources to provide optimal imaging and care for obese patients.

## Conflict of Interest

The authors declare no conflict of interest
